# Fracture neck of the talus with isolated talonavicular dislocation: A case report

**DOI:** 10.1097/MD.0000000000028073

**Published:** 2022-11-04

**Authors:** Amr Selim, Ali Zain Naqvi, Henry Magill, Jay Smith

**Affiliations:** a Cairo University Hospitals, Cairo, Egypt; b Homerton Hospital, London, UK; c Hillingdon Hospital, Uxbridge, UK; d Chelsea and Westminister Hospital, UK.

**Keywords:** Hawkins classification, talar neck, talonavicular dislocation

## Abstract

Talar neck fractures are injuries which frequently result in adverse outcomes or impairment of functional activity. Four groups were identified according to Hawkins classification and Canale modification. We present a female patient with a fractured neck of the talus associated with talonavicular dislocation, but intact tibiotalar and subtalar joints; an injury pattern not fulfilling the criteria described in the Hawkins classification.

**Patient concerns:**

A 46-year-old woman missed a step and fell down stairs with an immediate painful right ankle and inability to bear weight.

**Diagnosis:**

Talar neck fracture with an unusual isolated talonavicular dislocation.

**Interventions:**

Temporary closed reduction followed by open reduction and internal fixation were performed.

**Outcomes:**

The patient had excellent functional and radiological outcomes following surgical management.

**Conclusion:**

We discuss the management of this rare case in addition to a review of the current literature to provide the best evidence-based recommendations for this injury pattern.

## 1. Introduction

Talus fractures represent one of the most challenging injuries in orthopedic practice.^[1]^ It is the second most common tarsal bone to be broken and affects males more frequently.^[2]^ The talus plays a substantial role in ankle joint biomechanics, articulating with the tibia and fibula superiorly, calcaneus inferiorly, and navicular distally.^[3]^ Of all talar fractures, neck injuries are the most serious and may lead to long-standing implications on the patient’s lifestyle and function.^[4]^ The tenuous blood supply to the talar neck contributes to a high incidence of nonunion and avascular necrosis (AVN) that frequently occur with these injuries.^[5]^

Hawkins classification is the most widely accepted system used to identify the different patterns of talar neck fractures.^[5]^ Three groups were initially described: group 1 included undisplaced talar neck fractures, group 2 included subtalar dislocation, and group 3 included patients with subtalar and tibiotalar dislocations. Canale and Kelly added a fourth group involving cases of subtalar, tibiotalar, and talonavicular dislocation.^[6]^ We present a case of talar neck fracture associated with an isolated talonavicular dislocation, with intact subtalar and tibiotalar joints which failed to fit into any of the described groups. To the best of our knowledge, only four similar cases have been reported in the literature.

## 2. Case report

**Figure 1.**
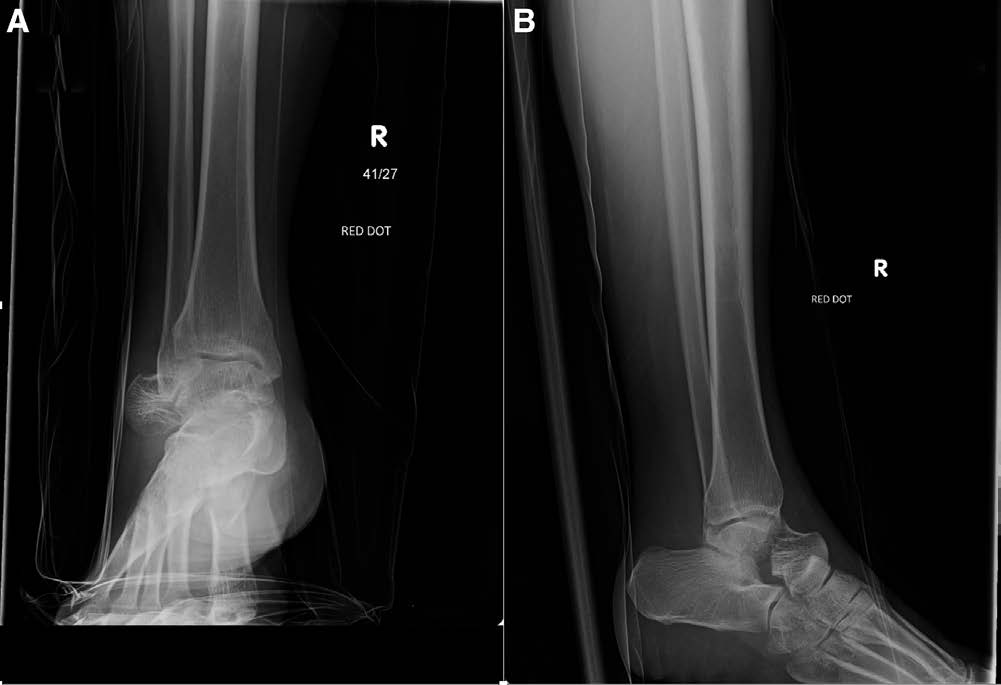
Ankle X-rays at the emergency department. (A) Anteroposterior (AP) and (B) Lateral (LAT) views showing the injury.

**Figure 2.**
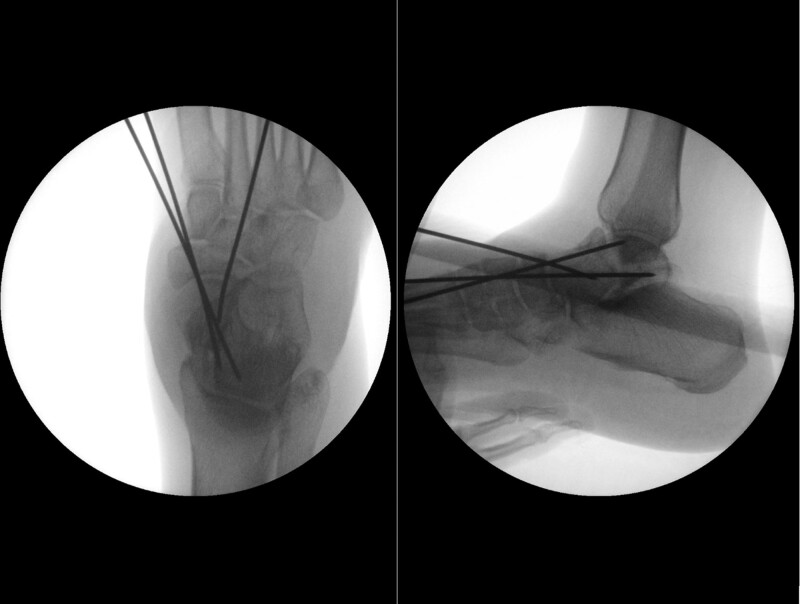
Intraoperative ankle X-rays. (A) AP and (B) LAT views confirming reduction of the talonavicular joint. AP = anteroposterior, LAT = lateral.

A 46-year-old woman missed a step and fell down stairs with an immediate painful right ankle and inability to bear weight. Examination revealed significant foot swelling, with a palpable bony mass just beneath the skin immediately distal and lateral to the ankle joint. Thorough examination of the distal neurovascular supply did not reveal any compromise, and the skin was intact with no visible wounds or abrasions. No other injuries were observed. Plain radiographs showed a displaced talar neck fracture with dislocation of the head and neck of the talonavicular joint. The subtalar and tibiotalar joints were intact (Fig. 1). The initial management in the accident and emergency department consisted of pain management in the form of 10 mg intravenous morphine in addition to application of a below-knee-back slab. The patient was kept nil by mouth and prepared for emergent closed reduction of the talus. The patient was transferred to the theater 3 hours after admission. Closed reduction of the talonavicular joint and temporary fixation with K-wires were planned to allow detailed imaging of the talus fracture pattern architecture. Closed reduction of the talonavicular joint was successfully achieved by direct pressure on the palpable dislocated head downward and medially, and then maintained by three 2 mm K-wires. Intraoperative imaging confirmed the reduction of the talonavicular joint (Fig. 2). The patient was placed in a below-knee-back slab with strict elevation and bed rest to allow soft tissues to settle.

**Figure 3.**
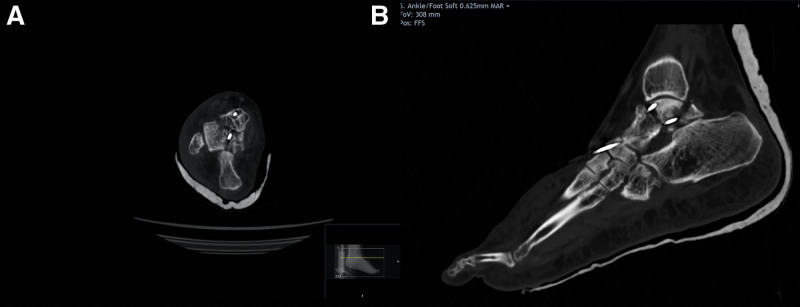
CT scan of the ankle. (A) Axial and (B) sagittal cuts delineating the fracture lines. CT = computed tomography.

**Figure 4.**
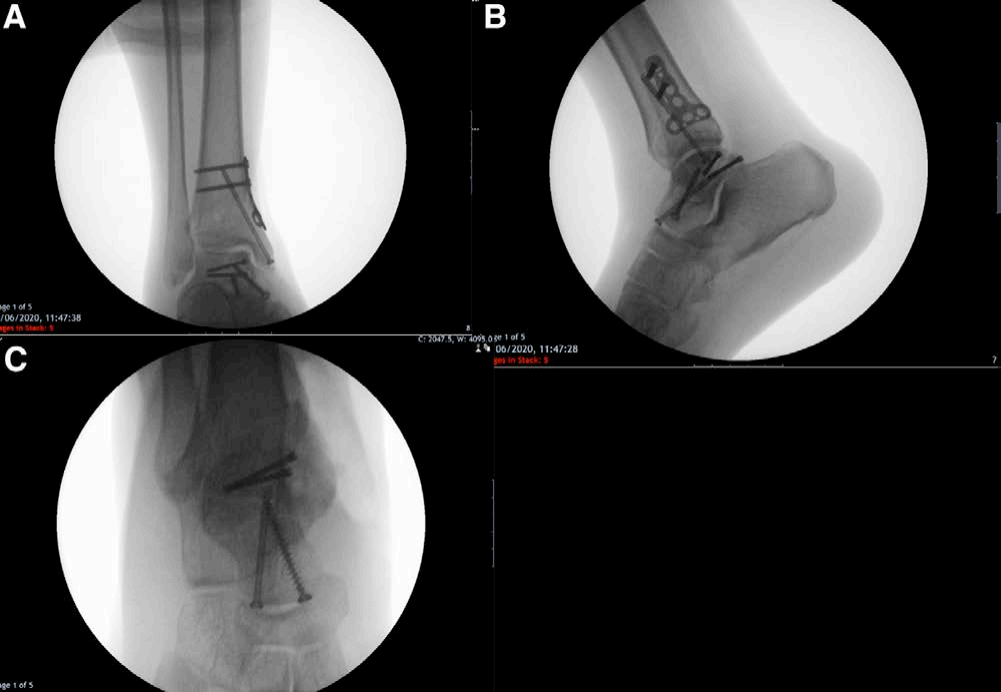
Intraoperative ankle X-rays. (A) AP, (B) LAT, and (C) Canale views confirming satisfactory open reduction and internal fixation of the talus. AP = anteroposterior, LAT = lateral.

On the following day, computed tomography of the foot and ankle was performed and revealed a fracture line passing through the talar neck in addition to another line passing through the posteromedial talar body dividing it into two fragments (Fig. 3). The patient was discussed in the foot and ankle multi-disciplinary team on the same day, and a decision of definitive fixation by open reduction and internal fixation through an anteromedial approach was advised. The second operation was performed two days after admission by a fellowship-trained foot and ankle surgeon once the soft tissue swelling was amenable. An anteromedial approach was utilized, and medial malleolar osteotomy was performed via a chevron cut. The comminuted posteromedial talar body was exposed, reduced, and fixed using two headless compression screws. The neurovascular bundles were identified and protected. The talar neck fracture was stabilized by lateral compression 4 mm cannulated screw, in addition to a medial cancellous non-compression 4 mm screw. Satisfactory position was confirmed with intraoperative imaging (anteroposterior, lateral, and canal views) (Fig. 4). The medial malleolar osteotomy was fixed with a buttress T plate and a 4 mm cannulated screw. Closure was performed in the layers. Non-weight-bearing mobilization in a boot was achieved for 6 weeks, followed by 2 weeks of partial weight-bearing.

At 6 weeks postoperatively, the plaster was removed, and the wounds had healed satisfactorily. Stiffness was present, and physiotherapy was requested. The range of movement was 5° dorsiflexion and 15° plantar flexion.

At the 12th postoperative week, the patient was mobilized with the aid of a walker boot without any pain. Confidence in walking unaided was still lacking, and the patient was under the care of the physiotherapists for ongoing rehabilitation and range of movement. Weight-bearing ankle X-rays were obtained at that point and showed union progression.

**Figure 5.**
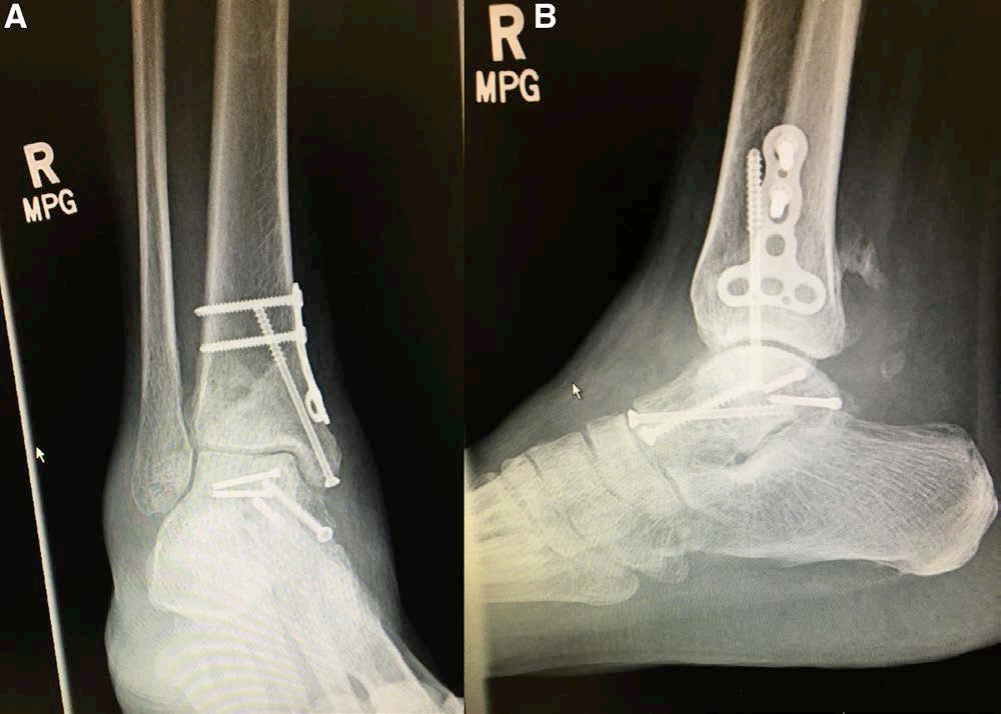
Weight-bearing ankle X-rays at the 1-year follow-up. (A) AP and (B) LAT views showing complete bony union with no signs of instability, AVN, or talar collapse. AP = anteroposterior, AVN = avascular necrosis, LAT = lateral.

At the 1-year follow-up, the patient completed the American Orthopaedic Foot and Ankle Society Ankle-Hindfoot Scale score, and new radiographs showed complete union of the fracture without any signs of instability, AVN, or talar collapse (Fig. 5). Extension and flexion at the tibiotalar joint were comparable to the contralateral side with inversion and eversion back to full (Supplemental Video, Supplemental Digital Content, http://links.lww.com/MD/H950).

## 3. Discussion and literature review

To the best of our knowledge, four similar cases have been reported in the literature.^[7–10]^ Pantazopoulos et al^[7]^ discussed a similar case in 1974. Open reduction through a medial approach and fixation using K-wires followed by plaster immobilization were utilized. Unfortunately, the patient developed infection and osteoarthritis; subsequently, pan-talar arthrodesis was performed with a net poor functional outcome. Khazim and Salo^[8]^ reported a 31-year-old man who fell from height and sustained a similar injury in addition to ipsilateral cuboid fracture, contralateral 1st metatarsophalangeal joint dislocation, and second lumbar vertebral fracture. On the same day, open reduction of the talus fracture through a medial approach and internal fixation using two 4.5 mm cannulated screws was performed. The screws were applied from the talar head in the anteromedial to posterolateral direction, followed by 6 weeks of knee cast immobilization. Three months postoperatively, the patient had only minimal foot discomfort for which a bone scan was performed and revealed AVN of the talar head without collapse. Veerappa et al discussed a 43-year-old man who had a road traffic accident and sustained the above-mentioned injury.^[9]^ Open reduction through a medial approach and internal fixation with two 4 mm cancellous screws was applied in a similar way to that reported by Khazim et al. Immobilisation in a below-knee cast was utilized for 2 months, and full weight-bearing was allowed at 3 months. The patient was regularly reviewed for 2 years with an eventless follow-up period. Kapoor and Patra^[10]^ described a 65-year-old man with a fractured neck of the talus with isolated talonavicular dislocation in addition to fracture of the posteromedial tubercule of the talus following a fall into a drain. In contrast, they utilized an anterolateral approach to access and reduce the talonavicular joint. Additionally, they only used one 3.5 Herbert screw in the anteromedial to posterolateral direction to fix the talus. They accessed the posteromedial talar tubercule through a posteromedial approach and fixed it with a 3.5 mm cancellous screw. A below-knee plaster was used for six weeks and full weight-bearing was initiated at the 18^th^ postoperative week. The patient was followed up for 24 months and, at this stage, the patient described foot pain while walking a long distance for which X-ray was performed and showed nonunion of the posteromedial tubercule. In our case, we followed a similar protocol for accessing the fracture through an anteromedial approach and fixing it using two 4.5 cannulated or cancellous screws. In contrast, we allowed partial and total weight-bearing earlier than what has been done by other authors. Functional scores were recorded using the American Orthopaedic Foot and Ankle Society Ankle-Hindfoot Scale at the 1-year mark with the patient scoring 92 out of a possible 100. The range of motion in the tibiotalar joint was excellent, with all movements returning to the normal range.

Collectively, most of the authors favored the anteromedial approach for accessing fractures with this configuration. Fixation using two 4 mm cannulated screws was the preferred method of fixation with placement of both screws in an anteromedial to posterolateral direction in previous cases. In our patient fixation, two 4 mm cannulated screws were placed in a convergent configuration. The protocol of plaster immobilization and non-weight-bearing was followed in all cases for 6 weeks, except by Pantazopoulos et al, who were immobilized for a prolonged period. Full weight-bearing was allowed 3 or 4 months after the operation, except in our case in which this was allowed in the 8th postoperative week. Three out of the 4 cases previously presented in the literature underwent early fixation and reported satisfactory outcomes with the remaining patient undergoing closed reduction and K-wire fixation as definitive surgery with reported poorer outcomes.

Early closed reduction and K-wire fixation allow for appropriate management of the soft tissue envelope and permit open reduction and internal fixation with adequate wound closure. According to the available evidence, we recommend early fixation of this injury, preferably by a foot and ankle surgeon. Staging surgical management to allow appropriate preoperative planning with computed tomography images or the availability of foot and ankle surgeons does not compromise the outcome. The anteromedial approach is thought to provide better exposure to access and reduce this fracture, and two 4 mm cannulated screws should be used whenever possible. We believe that the convergent configuration of the screws will be biomechanically superior to applying two parallel or divergent screws in the anteromedial to posterolateral direction. In addition, we believe that the early start of weight-bearing will result in a good functional outcome.

## Author contributions

**Data curation:** Amr Selim.

**Investigation:** Amr Selim, Ali Zain Naqvi.

**Supervision:** Ali Zain Naqvi, Jay Smith.

**Writing – original draft:** Amr Selim.

**Writing – review & editing:** Amr Selim, Ali Zain Naqvi, Henry Magill, Jay Smith.

## Supplementary Material


